# HPV8-E6 drives coordinated transcriptional and epigenetic reprogramming of keratinocytes

**DOI:** 10.1016/j.tvr.2026.200348

**Published:** 2026-07-13

**Authors:** Martin Hufbauer, Adnan Syed, Felix Bormann, Daniel Hasche, Manuel Rodríguez-Paredes, Pia Laine, Anni Honkimaa, Petri Auvinen, Eeva Auvinen, Baki Akgül

**Affiliations:** a Institute of Virology, National Reference Center for Papilloma- and Polyomaviruses, University of Cologne, Faculty of Medicine and University Hospital of Cologne, Cologne, Germany; b Bioinformatics.Expert UG, Berlin, Germany; c Division of Viral Transformation Mechanisms, German Cancer Research Center (DKFZ), Heidelberg, Germany; d Division of Epigenetics, DKFZ-ZMBH Alliance, German Cancer Research Center, Heidelberg, Germany; e DNA Sequencing and Genomics Laboratory, Institute of Biotechnology, University of Helsinki, Helsinki, Finland; f Department of Virology, Helsinki University Hospital and University of Helsinki, Helsinki, Finland

**Keywords:** Cutaneous squamous cell carcinoma, Beta human papillomavirus (betaHPV), HPV8 E6 and E7 oncoproteins, Keratinocyte transformation, Transcriptomic and epigenomic reprogramming, DNA methylation, RNA and miRNA sequencing

## Abstract

Beta human papillomaviruses (betaHPVs) act as cofactors in keratinocyte carcinogenesis, but how they reprogram host gene regulation remains unclear. Here, we combined transcriptomic, small RNA and epigenomic profiling to dissect the impact of betaHPV8 oncogene expression on keratinocytes. The viral E6 protein emerged as the major driver of host gene regulatory reprogramming, repressing epidermal differentiation programs, whereas E7 induced fewer, complementary changes. Small RNA sequencing revealed E6-dependent remodeling of the microRNA (miRNA) landscape, and integrative analyses linked miRNA regulation to reciprocal changes in target gene expression, indicating a role for epigenetic post-transcriptional regulation in the E6-driven phenotype. Whole-genome bisulfite sequencing showed widespread E6-dependent DNA methylation changes at promoters, enhancers, and gene bodies. Motif enrichment analysis showed that hypermethylation silenced nuclear receptor and developmental regulators, while hypomethylation was associated with enrichment of AP1 and STAT3 binding motifs. Analysis of clinical specimens further revealed that betaHPV DNA was enriched in actinic keratoses and cutaneous squamous cell carcinomas displaying stem cell-like methylation profiles, linking viral activity to progenitor-associated tumorigenesis. Together, our data define a multilayered mechanism by which HPV8 destabilizes keratinocyte lineage identity which may create a cellular state permissive for malignant progression.

## Highlights

• HPV8-E6 drives transcriptional and epigenetic reprogramming.
• E6 remodels miRNA networks and post-transcriptional regulation.
• DNA methylation changes expose AP-1 and STAT3 regulatory sites.
• betaHPV positivity associates with stem-like methylation in AK and cSCC.

## Graphical abstract

**Figure:**
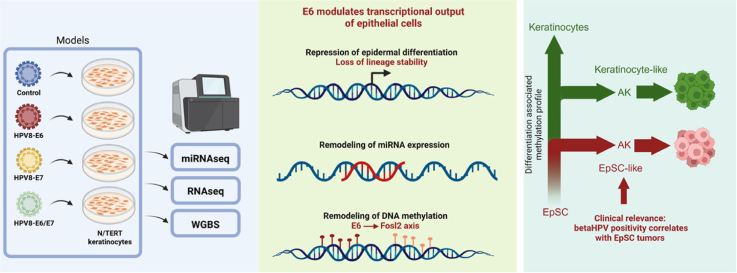

## 1 Introduction

Human papillomaviruses (HPV) of the genus Betapapillomavirus (betaHPV) are common, usually asymptomatic members of the human skin virome. Persistent infections, particularly those caused by HPV5 or HPV8, are nevertheless associated with cutaneous squamous cell carcinoma (cSCC) in patients with epidermodysplasia verruciformis (EV) and among immunosuppressed organ transplant recipients (OTRs) [1,2]. The contribution of betaHPV to cSCC development differs between EV patients and OTRs. In EV-associated cSCC, tumors typically retain high numbers of episomal viral genomes and virus protein expression, consistent with an ongoing role of the virus in tumor maintenance. In contrast, OTR-associated cSCCs retain very low or undetectable copy numbers of viral DNA and limited viral transcription, suggesting that betaHPV primarily contributes to tumor initiation rather than maintenance under immunosuppression [3,4]. Transgenic mouse models expressing HPV8 early genes under the control of the keratin-14 promoter have provided strong experimental evidence that this virus initiates and promotes skin tumorigenesis. These models have enabled functional dissection of the viral oncoproteins E2, E6, and E7 in keratinocyte reprogramming [[5], [6], [7], [8], [9]]. Latest results from transgenic mice reveal that HPV8-E6 induces STAT3 (Signal Transducer and Activator of Transcription 3) activity in the Lrig1 (Leucine-rich repeats and immunoglobulin-like domains protein 1) positive stem cell compartment to induce ΔNp63 expression, expansion of the stem cell compartment, and susceptibility to malignant transformation. These findings reveal that HPV8-E6 not only rewires transcriptional programs to create a permissive platform for tumor initiation and propagation but also attenuates tumor suppressing signaling to support oncogenic transformation [[10], [11], [12]]. HPV8-E6 also interferes with Notch signaling through interactions with p300 and MAML1 [[13], [14], [15]] thereby attenuating Hippo pathway activity and increasing cellular tolerance to genomic instability [16,17]. Overall, these functions reinforce a basal-like growth phenotype, promote survival under genotoxic stress, and define a tumor initiation phenotype [18,19].

Unlike E6, the E7 protein has potent effects on cell cycle regulation and invasion. Expression of HPV8-E7 induces aberrant keratin profiles, disrupts cell cycle checkpoints, and promotes invasion in organotypic skin cultures [9,[20], [21], [22], [23]]. Global transcriptomic and proteomic analyses revealed that E7 upregulates stress response genes and alters the expression and phosphorylation of key regulators of cell differentiation, DNA repair, and replication. Consistently, betaHPV oncoproteins were previously shown to interfere with RNA transcription, cell cycle control and cell differentiation in both 2D and 3D cell culture models [[24], [25], [26], [27], [28], [29]]. Collectively, HPV8 oncoproteins elicit multilayered transcriptional reprogramming, likely establishing their role as cofactors in UV-driven skin carcinogenesis. Yet, current information largely stems from array- and candidate gene-based analyses, however, comprehensive gene expression analyses of keratinocytes expressing HPV8-E6, E7 or both are lacking. Such an unbiased strategy is essential to assess the full spectrum of transcriptional regulation induced by the individual viral oncoproteins.

Beyond viral oncoproteins, epigenetic reprogramming has emerged as a hallmark of skin carcinogenesis. Epigenetic DNA methylation profiling of actinic keratoses (AK) and cSCC found that tumors can be subdivided into two classes with distinct origins [30]. One subtype retains methylation and chromatin accessibility patterns of basal keratinocytes (“Epi-SC-like”), consistent with stem cell origin and associated with higher invasiveness. The other subtype displays profiles consistent with keratinocyte differentiation (“keratinocyte-like”), with lower invasiveness but sustained proliferative capacity. Accordingly, benign warts caused by non-betaHPV display keratinocyte-like methylation profiles, in agreement with their low malignant potential [31].

Comprehensive transcriptomic and epigenomic characterization of keratinocytes expressing HPV8 oncogenes is still lacking. To address this gap, we combined RNA sequencing (RNAseq), small RNA sequencing and whole-genome bisulfite sequencing (WGBS) in keratinocytes expressing HPV8-E6, -E7, or -E6/E7. This approach allowed us to link transcriptional regulation with DNA methylation changes to provide a genome-wide landscape of how HPV8 oncoproteins rewire host gene expression. Our goal was to define how HPV8 alters host gene expression and DNA methylation to explore the hypothesis that betaHPV-induced epigenetic reprogramming establishes a permissive state favoring malignant progression.

## 2 Materials and methods

### 2.1 Cell lines and treatments

The human keratinocyte telomerase reverse transcriptase-immortalized (h/TERT-immortalized) cell line N/TERT derived from clinically normal foreskin tissue (kindly provided by James Rheinwald, Harvard Medical School, Boston, MA, USA) [32] was cultivated in KGM-Gold (Lonza, Cologne, Germany). The production of recombinant pLXSN-based retroviruses coding for HPV8-E6, -E7 or -E6/E7, their subsequent transduction into N/TERTs, and the selection of stable, 100% transduced clones was performed as described previously [33]. Cells transduced with pLXSN alone served as controls. Cells were routinely screened for mycoplasma contamination using PCR-based assays [34]. A retrospective analysis identified mycoplasma contamination in the HPV8-E6 RNAseq samples after completion of the sequencing experiments. Independent validation experiments were therefore performed using newly generated mycoplasma-free HPV8-E6 keratinocytes.

### 2.2 RNA isolation and RNAseq

Total RNA was isolated from cells using the RNeasy kit, and DNase digestion was performed on a column using RNase-free DNase according to the manufacturer's instructions (Qiagen, Hilden, Germany). Total RNA (240-488 ng) was used for library construction. RNA library preparation and bulk RNA sequencing (RNAseq) after removing rRNA molecules was performed using a TruSeq Stranded RNA HT kit including ribozero H/M/R (Illumina). Sequencing was performed with NextSeq500 (Illumina) using a single end 75 bp mode. Barcodes were selected using the BARCOSEL software [35].

### 2.3 RNAseq data processing and differential expression analysis

Initial quality control of raw FASTQ files was performed using FastQC (v0.11.9). Reads were trimmed from the 5′-ends using the program Trimmomatic (v.0.39) with the parameter HEADCROP:15. Subsequently, the trimmed reads were re-evaluated with FastQC to verify improved read quality. Trimmed reads were aligned to the human reference genome (*Homo sapiens*, GRCh38, Ensembl release 112 (https://ftp.ensembl.org/pub/release-112/fasta/homo_sapiens/dna/Homo_sapiens.GRCh38.dna.primary_assembly.fa.gz)) using STAR (v2.7.11b) with key parameters including outFilterMultimapNmax 20, outFilterMismatchNmax 10, alignIntronMax 500000, sjdbScore 2, and minimum alignment and score fractions of 0.33. Output BAM files were sorted by genomic coordinate using the STAR parameter outSAMtype BAM SortedByCoordinate. Gene-level read quantification was performed with featureCounts from the Subread package (v2.1.1) using the parameter -s 2, Homo_sapiens.GRCh38.112.gtf as reference (https://ftp.ensembl.org/pub/release-112/gtf/homo_sapiens/) and the sorted bam files as input. The resulting raw count matrix was used as an input for differential gene expression analysis using DESeq2 (v1.48.1). Differential expression was assessed by comparing 3 replicates of group E6, E7 or E6/E7 to 3 replicate control samples. Genes with an absolute log2FC of equal or greater than 1 and an adjusted p-value below 0.05 were considered significantly differentially expressed. Additionally, StringTie (v3.0.0, parameters: -e and --rf) was used to estimate transcript and gene-level expression in TPM (Transcripts per Million).

### 2.4 MicroRNA sequencing

The total RNA preparations used for mRNA sequencing were also used as material for microRNA (miRNA) sequencing. Libraries were constructed using Illumina TruSeq Small RNA Library Preparation Kit (Illumina Inc., San Diego, California) from 100 ng of total RNA as starting material. Libraries were purified as instructed by the manufacturer and pooled. The pool was size-selected using BluePippin (Sage Science, Beverly, Massachusetts). The barcodes for libraries were selected using the BARCOSEL tool [35]. Sequencing was performed with a NextSeq500 instrument (Illumina inc.) and 75bp single end kit.

### 2.5 miRNA analysis

Raw small RNA sequencing reads were analyzed using miRge3.0 with the human reference based on miRGeneDB [36]. Adapter trimming, quality filtering, read collapsing, and alignment were performed automatically within miRge3. Reads were classified into RNA categories and used to generate summary bar plots describing library composition. Differential expression analysis was conducted using the integrated DESeq2 workflow implemented in miRge3 and a predefined sample metadata file. The DESeq2 dataset object was exported and processed in R (version 4.5.0) and used for data visualization. miRNA interaction network analysis was performed using miRNet 2.0, a comprehensive web-based platform for integrative analysis and visualization of miRNA-centered regulatory networks [37]. A list of differentially expressed miRNAs was uploaded to miRNet using the *Homo sapiens* reference dataset. Target genes were based on miRTarBase v9.0. The network analysis was filtered for differentially expressed target genes based on RNAseq data.

### 2.6 Data visualization and exploration

Visualization and further exploratory efforts were made using R statistics version 4.5.0. Principal component analysis (PCA) was performed with the R package FactoMineR (version 2.12) using the normalized counts after DEseq2 analysis (DESeq2 version 1.48.1). Volcano plots were generated in R using ggplot2 (version 3.5.2) by plotting DESeq2 log2 fold changes against −log10 adjusted p-values to highlight significantly differentially expressed protein coding genes or miRNAs. Venn Diagrams were created using the R package VennDiagram (version 1.7.3). IDEP 2.20.2 (http://bioinformatics.sdstate.edu/idep/) was used to calculate GO enrichment from raw counts. Ingenuity Pathway Analysis (IPA; Qiagen) Comparison analysis function was utilized to compare and visualize differentially expressed canonical pathways based on RNAseq data sets HPV8-E6, -E7 and -E6/E7. IPA MicroRNA Target Filter function was utilized to visualize differentially expressed HPV-E6 miRNAs with experimentally validated human mRNA targets including only the targets differentially expressed in the HPV8-E6 RNAseq dataset.

### 2.7 DNA isolation and whole genome bisulfite sequencing (WGBS)

Genomic DNA was extracted using proteinase K lysis and purification by the QIAamp DNA Mini Kit (Qiagen). DNA (600 ng) was treated with bisulfite, except for samples used as treatment controls, followed by purification and shearing to suitable fragment lengths using Bioruptor NGS (Diagenode). Adapters were ligated to the fragment ends followed by 15 PCR cycles. Final libraries were purified and size measured with Fragment Analyzer (Agilent) and size-selected (peak ca. 400 bp). Sequencing was performed with NovaSeq6000 (Illumina) using paired end 150 + 150 bp mode. Barcodes were selected using the BARCOSEL software [35].

### 2.8 WGBS data processing and analysis

WGBS data were analyzed using the Snakemake-based pipeline wg-blimp (v.0.9.10). Initial quality control of the raw data fastq.gz files was performed using FastQC (v.0.11.9). The reads were then aligned to the human reference genome (GRCh38) (https://ftp.ensembl.org/pub/release-112/fasta/homo_sapiens/dna/Homo_sapiens.GRCh38.dna.primary_assembly.fa.gz using BWA-Meth (v0.2.0). The resulting bam files were sorted by genomic coordinates using samtools (v1.15.1). PCR duplicates were identified and marked using picard MarkDuplicates (v.2.26.5). Quality control metrics were generated as part of the wg-blimp pipeline. Cytosine methylation levels in CpG and non-CpG contexts were extracted from aligned bam files using MethylDackel (v0.4.0), generating context-specific bedGraph files for downstream analyses. Bisulfite conversion efficiency was estimated from CHH and CHG contexts by calculating one minus the fraction of methylated calls at sites with coverage ≥5. CpG site coverage distributions were derived from CpG bedGraph files by calculating per-site coverage as the sum of methylated and unmethylated reads and visualized as empirical cumulative distribution functions. Global CpG methylation distributions were generated by counting integer methylation percentages (0–100%) at CpG sites with coverage ≥5 and converting counts to relative frequencies. For genome-wide exploratory analysis, CpG methylation values were converted to bigWig format and summarized in non-overlapping 100 kb bins using deepTools (v3.5.6). PCA was performed in R (v4.5.0) using FactoMineR (v2.12) and visualized with ggplot2 (v3.5.2). Genome-wide methylation segmentation into unmethylated regions (UMRs), low-methylated regions (LMRs), and partially methylated domains (PMDs) was performed as part of the wg-blimp pipeline. The total genomic coverage of UMRs, LMRs, and PMDs was quantified per sample by summing region lengths from the segmentation output and expressing them as fractions of the genome. Mean CpG methylation levels across genomic features were quantified by intersecting CpG bedGraph files with genomic feature annotations and retaining CpG sites with total coverage ≥5 reads. Mean methylation per feature interval was calculated using bedtools map with the mean operator. Group differences were assessed using two-sided Wilcoxon rank-sum tests with Benjamini-Hochberg correction. Differentially methylated regions (DMRs) were identified using bsseq (v1.30.0) applying the BSmooth.tstat and dmrFinder functions under default settings. Smoothed methylation profiles were calculated across the genome and resulting DMRs were filtered to retain regions showing a minimum absolute methylation difference of 0.15.

The sequencing data generated in this study have been deposited in the European Nucleotide Archive (ENA) under accession number PRJEB108515.

### 2.9 Transcription factor binding site analysis and integration with RNAseq data

DMRs were annotated using GenomicRanges by overlapping them with genomic features (promoters (TSS200, TSS1500, 5′UTRs, 1stExon), gene bodies, enhancers and 3′UTR) and corresponding genes from the GRCh38.112 transcriptome (https://ftp.ensembl.org/pub/release-112/gtf/homo_sapiens/). Annotated DMRs were then stratified by genomic feature and direction of methylation change, exported as BED files, and converted to fasta files using bedtools (version 2.25.0). Transcription factor binding site (TFBS) analyses were performed with the HOMER function findMotifs.pl (version 5.1) using the vertebrate motif database.

For each DMR set, background sequences were generated with the HOMER function homer2 bg, using the human reference genome hg38 (http://homer.ucsd.edu/homer/data/genomes/hg38.v7.0.zip) and matched dinucleotide composition (homer2 bg -ikmer 2). The top 20 enriched TFBSs were selected and the corresponding transcription factor TPM expression values were extracted from the RNAseq dataset. Only TFs with an expression level of at least 1 were considered suitable candidates for methylation specific epigenetic regulation. Next, the original DMRs were linked to these TFs by scanning their genomic coordinates with TF motif files using annotatePeaks.pl (hg38, -noann). TF-associated DMRs were identified by matching motif hits to the corresponding feature-annotated DMR sets. Finally, the TF-regulated genes were identified by extracting gene annotation from TF specific DMRs. To assess potential epigenetic regulation, TPM expression levels of TF-regulated genes were compared to controls, and genes showing a statistically significant difference (padj <0.05) were considered epigenetically regulated. Only genes showing expression changes consistent with the predicted direction of regulation (up or down) were retained for downstream analyses. Gene Ontology (GO) enrichment analysis was then performed using a locally adapted version of the shinyGO pipeline. Input gene sets consisted of the epigenetically regulated genes associated with DMRs in promoters, enhancers, or gene bodies, with all DMR-associated genes used as the background set. Enrichment for GO Biological Process (GOBP) categories was assessed using a hypergeometric test, and p-values were adjusted by the Benjamini-Hochberg method to obtain False Discovery Rates (FDR). Only GO terms with FDR ≤0.05 were considered significant.

### 2.10 Quantitative PCR following reverse transcription (RT-qPCR)

One microgram of total RNA was reverse transcribed using the Omniscript RT kit (Qiagen) with 10 μM random nonamers (TIB MOLBIOL, Berlin, Germany) and 1 μM oligo(dT23) primer (Sigma), as well as 10 units of RNase inhibitor (Fermentas, St. Leon-Rot, Germany). Quantitative PCR (qPCR) was performed using the GoTaq-qPCR SYBRGreen master mix (A6002, Promega, Walldorf, Germany) and the Light-Cycler system (Roche). Samples were analyzed in duplicate together with a cDNA dilution series, which was used to generate a standard curve. Mean values were used for the calculation of expression ratios using the Pfaffl equation [38]. The mRNA expression levels of target genes were normalized to the mRNA levels of hypoxanthine phosphoribosyltransferase 1 (HPRT1). The primers used in this study are listed in Supplementary Table 1.

### 2.11 BetaHPV genotyping of AK and cSCC

AK and cSCC samples were obtained as punch biopsies (diameter 4 mm) at the Charité University Hospital (Berlin, Germany). The study was approved by the local ethics committee at the Charité, University Hospital, Berlin, Germany (number Si. 248). The epidermal parts were separated from the dermal parts of the punch biopsies by heat split (56 °C for 2 min) and careful manual dissection. DNA was isolated using the QIAamp DNA Investigator Kit (Qiagen). These samples were previously described [30]. Extracted DNA was subjected to a pan-betaHPV PCR to identify positive samples which were further subjected to a reverse hybridization assay (RHA) based skin (beta) HPV genotyping test (Labo Bio-medical Products B.V.) that distinguishes between 25 individual betaHPV genotypes [39].

## 3 Results

### 3.1 E6 dominates HPV8-induced transcriptional reprogramming

**Fig. 1:**
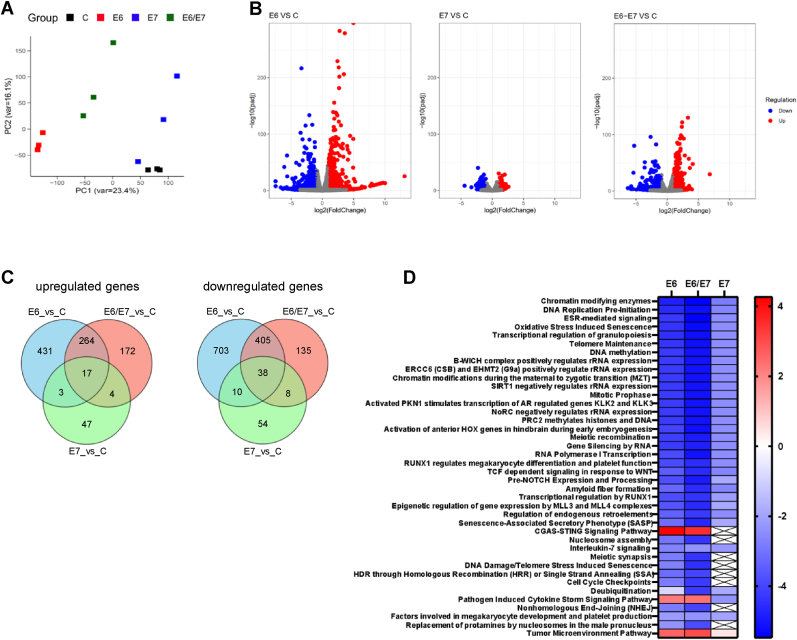
Differentially expressed genes upon HPV8 oncogene expression. (A) Principal component analysis (PCA) was performed on RNAseq data from HPV8-E6, -E7 or -E6/E7 expressing N/TERT keratinocytes. (B) Volcano plots showing differentially expressed genes in N/TERT cells expressing HPV8-E6 (E6_vs_C), -E7 (E7_vs_C), or -E6/E7 (E6/E7_vs_C) compared to control cells containing the empty vector as determined by RNAseq analysis. Each dot represents one gene plotted according to log2 fold change (x-axis) and –log10 adjusted p-value (y-axis). Genes meeting the significance criteria (|log2FC| > 1 and padj <0.05) are highlighted. (C) Venn diagrams depicting significantly upregulated (left) or downregulated (right) genes (|log2FC| > 1; padj <0.05) in HPV8 positive keratinocytes compared to N/TERT control keratinocytes for each comparison (E6_vs_C, E7_vs_C, E6/E7_vs_C). Numbers indicate unique and overlapping sets of deregulated genes. (D) Ingenuity Pathway Analysis (IPA) comparison of canonical pathways regulated by HPV8 oncoproteins. IPA comparison revealed that E6 and E6/E7 cells share nearly identical pathway signatures, characterized by broad suppression of signaling pathways. Only three pathways (marked in red) were predicted to be activated in both conditions. E7 alone showed minimal pathway changes, indicating that E6 is the principal driver of pathway-level reprogramming, with E7 contributing only minor additional effects.

To explore the impact of HPV8 oncogene expression on keratinocytes, we generated N/TERT cells expressing HPV8-E6, -E7, or both E6 and E7 proteins (-E6/E7) and subjected them to transcriptomic analysis (Fig. 1A). RNAseq analysis revealed transcriptional reprogramming in all HPV8 oncogene-expressing cells (Supplementary Table 2). In E6-expressing cells, the volcano plot displays a broad and dense distribution of differentially regulated genes (DEG) on both sides of the x-axis, resulting in the upregulation of 715 and downregulation of 1156 genes (padj <0.05 and log2-fold change >1 or < -1). In contrast, the volcano plot of E7-expressing cells shows only a small number of genes passing the thresholds (71 up, 110 down), indicating limited regulation of transcription by E7. The E6/E7 volcano plot closely resembles the E6 profile but with a moderately reduced number of significantly regulated genes (457 up, 586 down), suggesting that co-expression with E7 does not amplify global transcriptional disruption but rather modulates the E6-induced program. The strong visual similarity between the E6 and E6/E7 volcano plots reinforces the conclusion that E6 is the principal driver of transcriptomic reprogramming, whereas E7 alone exerts only limited global effects (Fig. 1B, C). IPA Canonical Pathways comparison analysis showed that E6 and E6/E7 cells display nearly identical canonical pathway signatures, dominated by strong inhibition of innate immune and antiviral pathways, while E7 alone shows only minimal effects. Notably, only three pathways, namely “CGAS-STING signaling”, “Pathogen Induced Cytokine Storm Signaling Pathway” and “Tumor Microenvironment pathway”, were predicted to be activated in E6 and E6/E7 expressing cells which otherwise shared the same overall pattern of broad pathway suppression. These findings indicate that E6 is the principal driver of pathway-level changes, with only minor additional activation events occurring when E6 and E7 are co-expressed (Fig. 1D). Together, volcano plots and pathway data identify E6 as the principal driver of transcriptional reprogramming in keratinocytes. By contrast, E7 induces comparatively modest and distinct transcriptional changes that complement but do not override the dominant E6-driven program.

To determine whether the reduced number of differentially expressed genes in E6/E7 cells compared to E6 cells could be explained by differences in oncogene expression levels, we examined E6 and E7 transcript abundance in the RNAseq datasets. E6 expression levels were comparable between E6 and E6/E7 cells, while E7 transcripts were robustly expressed in both E7 and E6/E7 conditions (Supplementary Fig. 1). These findings indicate that the attenuated number of transcriptional changes observed in E6/E7 cells is not attributable to reduced oncogene expression levels but rather reflects modulation of the E6-driven transcriptional program in the presence of E7.

### 3.2 Gene Ontology analyses of HPV8 oncogene-expressing keratinocytes uncover distinct and partially complementary transcriptional programs

Gene Ontology (GO) pathway analysis revealed distinct patterns of transcriptional reprogramming (Supplementary Table 3). In HPV8-E6-expressing keratinocytes, upregulated pathways were predominantly associated with immune and inflammatory responses, signal transduction, and cell cycle-related processes. Downregulated pathways were dominated by metabolic and biosynthetic processes, together with pathways related to developmental programs and cell differentiation, such as “Epidermis development” and “Epithelial cell differentiation” (Table 1). In contrast, E7 expression showed a different trend, characterized by suppression of immune pathways and activation of developmental and differentiation programs, with only minor effects on metabolism. In E6/E7 co-expressing keratinocytes, the transcriptional profile largely resembled the E6 pattern, indicating that E6 dominates transcriptional reprogramming and drives a cellular state characterized by reduced differentiation which may contribute to the initiation of epidermal transformation.

**Table 1:** Gene Ontology enrichment analysis of deregulated genes in HPV8-E6 oncogene-expressing keratinocytes. Gene Ontology (GO) enrichment was performed using iDEP 2.20.2 with an FDR cutoff of ≤0.05. Significantly enriched categories include processes related to epidermal differentiation, immune regulation, and chromatin organization (complete list, see Supplementary Table 3).

group	FDR	nGenes	Pathway size	Fold enriched	Pathway
Down	6.8E-02	5	30	6.1	Cell-cell adhesion mediated by cadherin
Down	8.0E-02	6	88	4.7	Keratinization
Down	1.2E-02	29	414	2.4	Epidermis development
Down	2.6E-02	24	335	2.3	Skin development
Up	1.0E-09	97	1796	2.1	Cell adhesion
Down	2.7E-02	42	800	1.8	Epithelial cell differentiation
Down	1.6E-02	67	1314	1.6	Epithelium development
Down	5.0E-03	103	2165	1.5	Tissue development

**Fig. 2:**
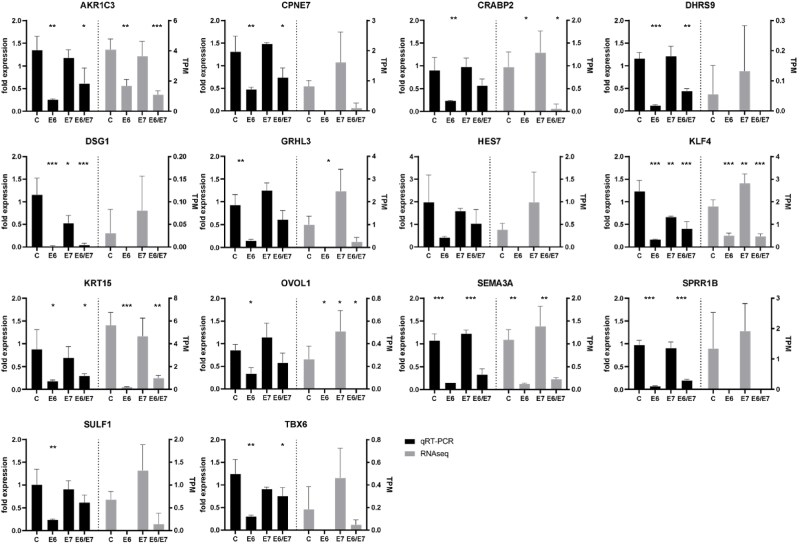
Validation of RNAseq results by RT-qPCR. Comparison of RNAseq and RT-qPCR results for selected genes associated with the GO term “epidermal development”. Expression changes measured by RT-qPCR closely mirrored RNAseq results, confirming the robustness of the transcriptomic analysis. Shown are representative genes involved in keratinocyte differentiation, cytoskeletal organization, epidermal barrier formation, and transcriptional control of epithelial identity. Bars represent mean expression ± SD of three biological replicates measured in duplicate. Asterisks indicate significance levels calculated relative to vector controls (C) (∗p < 0.05; ∗∗p < 0.01; ∗∗∗p < 0.001). Black bars: fold expression changes measured by RT-qPCR. Gray bars represent transcripts per million (TPM) measured by RNAseq.

To further substantiate the GO categories “Epidermis development” and “Epithelial cell differentiation,” we selected representative genes for validation (Fig. 2). RT-qPCR confirmed consistent downregulation of AKR1C, CPNE7, CRABP2, DHRS9, DSG1, GRHL3, HES7, KLF4, KRT15, OVOL1, SEMA3A, SPRR1B, SULF1, and TBX6 in HPV8-E6 and -E6/E7-expressing keratinocytes, but not in cells expressing E7 alone. These genes encompass key regulators of keratinocyte lineage and barrier formation, including TFs (OVOL1, GRHL3, KLF4), structural and adhesion molecules (DSG1, SPRR1B, KRT15), and mediators of retinoic acid metabolism (CRABP2, DHRS9, AKR1C). Their concerted suppression underscores the dominant role of E6 in repressing epidermal differentiation programs.

During data analysis, a mycoplasma infection was detected in the E6-expressing cells. Because mycoplasma contamination has been reported to affect cell morphology, metabolism, and growth [40], we repeated the RT-qPCR analyses using mycoplasma-free E6-expressing cells and confirmed that the transcriptional patterns remained unaffected and that the conclusions of the study were unchanged. Notably, the transcriptional changes observed in E6-expressing cells were highly consistent with those identified in constantly mycoplasma-negative E6/E7-expressing cells, further supporting the robustness of our findings (Supplementary Fig. 2).

### 3.3 HPV8-E6 drives remodeling of the keratinocyte miRNA landscape

**Fig. 3:**
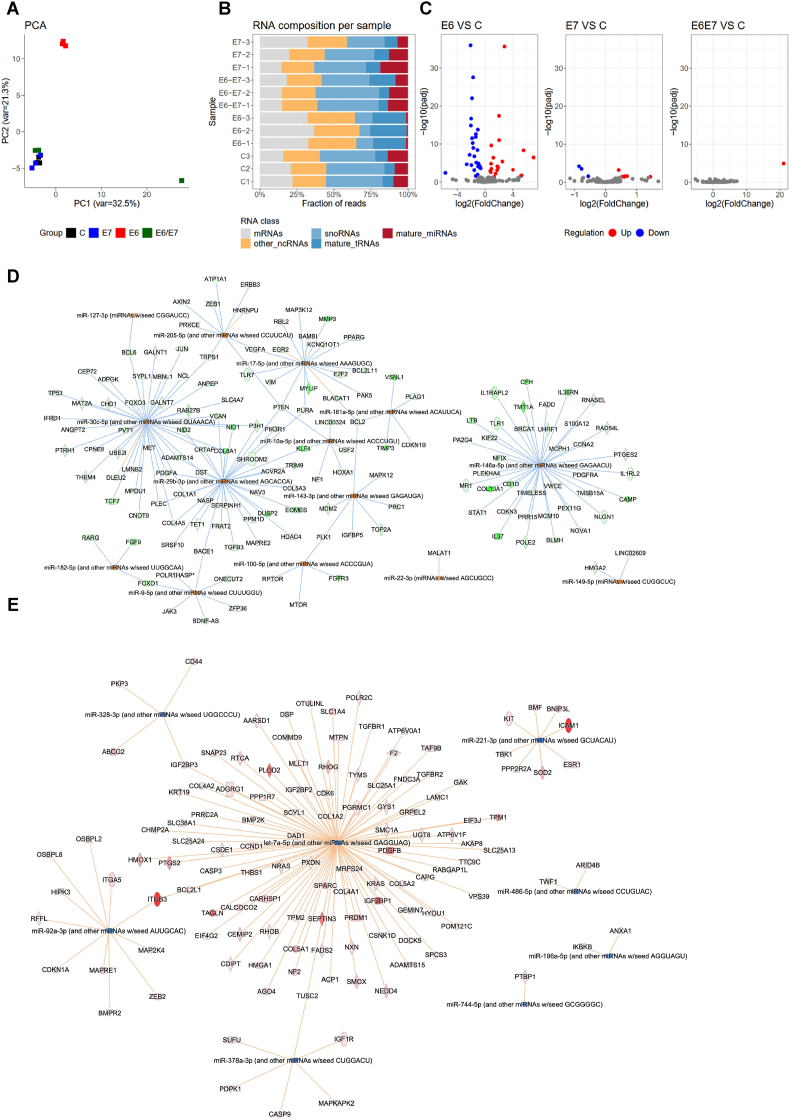
HPV8-E6 drives remodeling of the miRNA landscape and miRNA–mRNA regulatory networks. (A) PCA of small RNA sequencing data from N/TERT keratinocytes expressing HPV8-E6, -E7 or -E6/E7 compared with vector control cells. (B) RNA class composition of small RNA sequencing. Bar plots show the relative fraction of reads mapping to major RNA categories, including miRNAs, mature miRNAs, mature tRNAs, snoRNAs, other non-coding RNAs, and mRNAs. (C) Volcano plots showing differential miRNA expression. (D) Integrative miRNA - mRNA interaction network linking upregulated miRNAs to downregulated target genes and (E) downregulated miRNAs to upregulated target genes identified in the HPV8-E6 RNAseq dataset.

To assess whether HPV8-induced transcriptional changes extend to the small RNA level and involve post-transcriptional regulation, we performed small RNA sequencing in cells expressing HPV8-E6, -E7, or -E6/E7 (Fig. 3A). To further evaluate whether HPV8 oncogene expression globally affects small RNA biogenesis, we examined the RNA class composition across all samples (Fig. 3B). The relative fraction of reads for “miRNAs”, “mature miRNAs”, “mature tRNAs”, “snoRNAs”, and “other non-coding RNA” species were largely consistent between control, E7, and E6/E7 groups. In contrast, E6-expressing cells displayed an increase in reads mapping to “mRNAs” accompanied by a relative decrease in “mature miRNA” reads. This altered RNA class distribution suggests selective remodeling of the small RNA landscape in response to E6 expression. The Volcano plots show that E6 expression induced the strongest and most widespread miRNA deregulation, whereas E7 and E6/E7 caused comparatively minor changes (Fig. 3C). Significantly upregulated and downregulated miRNAs (padj <0.05; Supplementary Table 4) were subsequently subjected to integrative experimentally validated miRNA target enrichment analysis based on HPV8-E6 DEGs using IPA MicroRNA Target Filter analysis to identify potential regulatory networks. Regulatory clusters were centered around miR-143-3p, miR-29b-3p, miR-30c-5p, miR-181a-5p, miR-100-5p, miR-9-5p, miR-182-5p, and miR-22-3p (Fig. 3D). Conversely, a prominent let-7a-5p hub dominated among downregulated miRNAs (Fig. 3E). These reciprocal interaction networks demonstrate that HPV8-E6 reshapes gene expression programs through coordinated transcriptional changes and selective miRNA-mediated post-transcriptional regulation.

### 3.4 HPV8 oncoproteins reshape the keratinocyte methylome

**Fig. 4:**
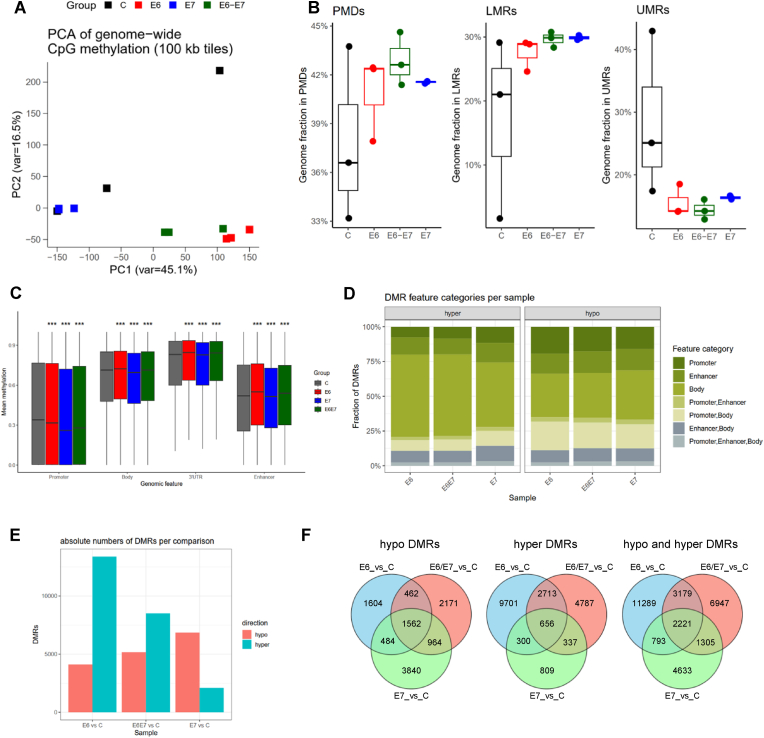
DMRs regulated by HPV8. WGBS of N/TERT keratinocytes expressing HPV8- E6, -E7, or -E6/E7 identified extensive hypo- and hypermethylated regions compared to controls. (A) PCA was performed on WGBS data. (B) Changes of partially methylated domains (PMDs), low-methylated regions (LMRs) and unmethylated regions (UMRs) in oncogene-positive cells. Genomic localization of total DMRs (C) and hyper- or hypomethylated genome regions (D). (E) Changes in absolute numbers of DMRs. (F) Venn diagrams show hypomethylated DMRs (left), hypermethylated DMRs (middle), and the combined set of all DMRs (right) for each comparison (E6_vs_C, E7_vs_C, E6/E7_vs_C). Diagrams are constructed from merged genomic intervals, which are generated independently for the hypomethylated, hypermethylated and combined DMR sets. As a result, their overlap counts are not additive, because partially overlapping DMRs collapse differently upon merging.

To investigate whether transcriptional changes are also accompanied by alterations in DNA methylation, we next performed whole-genome bisulfite sequencing (WGBS) across all oncogene conditions (Fig. 4A). Sequencing quality metrics, including bisulfite conversion efficiency, CpG coverage, and global methylation distributions, confirmed the high quality and comparability of all libraries (Supplementary Fig. 3). Genome-wide methylation profiling revealed broad alterations of the epigenetic landscape in HPV8-expressing keratinocytes. Compared to controls, we observed an increased genomic coverage of partially methylated domains (PMDs) and low-methylated regions (LMRs), accompanied by a reduction in unmethylated regions (UMRs) (Fig. 4B). In addition, mean CpG methylation levels were consistently decreased across promoters (defined as the union of 5′UTR, first exon, TSS1500, and TSS200 regions, consistent with Illumina Infinium methylation annotation [41]), enhancers, gene bodies, and 3′UTRs compared to ChromHMM segmentation data from normal human epidermal keratinocytes [42]), indicating a global redistribution of DNA methylation at functional genomic elements (Fig. 4C). Stratification of DMRs by genomic context demonstrated that methylation changes were broadly distributed across promoters, enhancers, gene bodies, and intergenic regions in all oncogene conditions (Fig. 4D).

Quantification of absolute DMR numbers further revealed that E6 induced the highest number of methylation alterations, followed by E6/E7, whereas E7 alone exhibited markedly fewer DMRs (Fig. 4E), underscoring the dominant epigenetic impact of E6. Consistent with these global changes, we identified a high number of differentially methylated regions (DMRs) with an absolute mean methylation difference of ≥0.15 relative to controls. These DMRs comprised both hyper- and hypomethylated sites annotated to promoters, enhancers, and gene bodies. For downstream analyses, DMRs were categorized as E6/hyper, E6/hypo, E7/hyper, E7/hypo, E6/E7/hyper, and E6/E7/hypo, where hypermethylation denotes increased methylation and hypomethylation denotes relative loss of methylation in HPV8-expressing cells compared to controls. A substantial overlap between E6 and E6/E7 DMRs was seen, while E7 shared only a limited fraction and contributed a distinct subset of unique regions (Fig. 4F; Supplementary Table 5). Direct comparison of hyper- and hypomethylated DMRs across conditions further highlighted the strong concordance between E6 and E6/E7 methylation landscapes, whereas E7 displayed fewer and more selective alterations, mirroring the dominant role of E6 observed at the transcriptomic level. Taken together, these data demonstrate that HPV8 oncoproteins extensively and systematically reshape the keratinocyte methylome at functionally relevant regulatory elements, providing an additional epigenetic layer that likely reinforces and stabilizes the observed transcriptional reprogramming.

### 3.5 HPV8 oncoproteins reprogram transcription factor binding site landscapes via differential DNA methylation

**Fig. 5:**
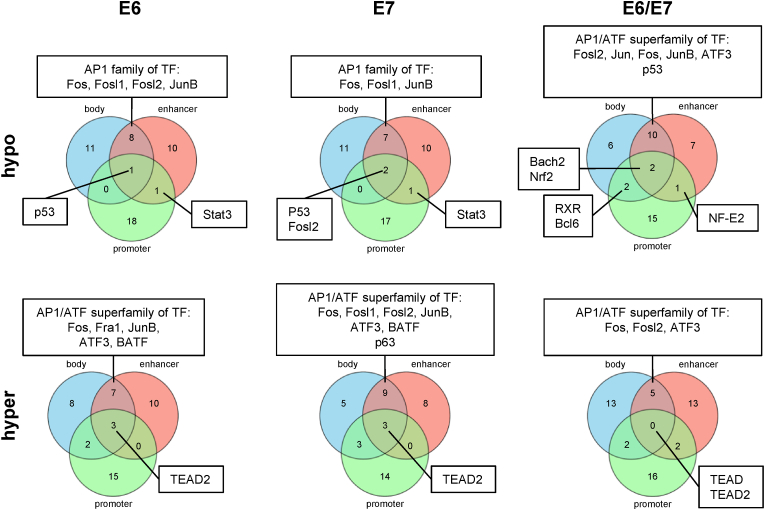
Motif enrichment in DMRs in HPV8 oncogene-expressing keratinocytes. Motif enrichment analysis of WGBS-defined DMRs in HPV8-E6, -E7 or -E6/E7 expressing N/TERT keratinocytes revealed widespread remodeling of TF binding landscapes across gene bodies, enhancers, and promoters. AP1/ATF motifs were recurrently enriched in both hypo- and hypermethylated regions, indicating redistribution of these regulatory networks. Non-overlapping motifs point to selective silencing or exposure of regulatory networks.

Next, we performed HOMER TF binding motif enrichment analyses in the different DMR sets (Supplementary Table 6). The top 20 TFs with enriched binding sites were selected for a comparison of hyper- and hypomethylated motifs across gene bodies, enhancers, and promoters. The analysis revealed that gene bodies and enhancers share a substantial number of enriched TF binding sites, whereas promoters exhibit fewer shared motifs and more condition-specific enrichment patterns. AP1/ATF binding sites were detected in both hyper- and hypomethylated regions under all HPV oncogene-expression conditions, indicating a redistribution of this regulatory network rather than a unidirectional gain or loss (Fig. 5).

**Fig. 6:**
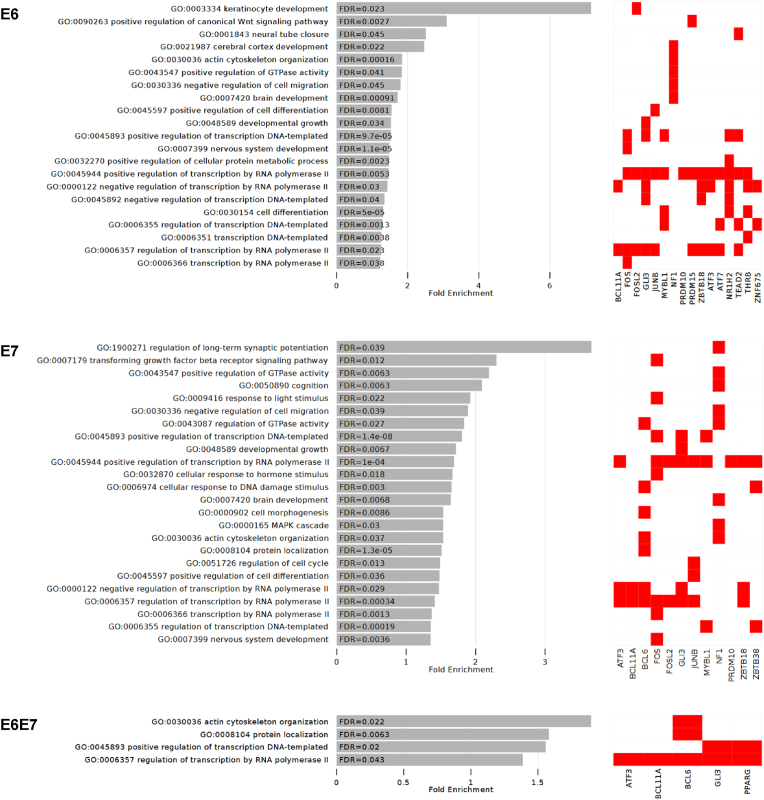
Transcription factor-GO term associations derived from DMR-linked and expressed TFs in HPV8 oncogene-expressing keratinocytes. Intersection of WGBS-derived DMR motifs with RNAseq data identified TFs that were both expressed and associated with DMRs, representing functional regulators of methylation-driven transcriptional changes. GO-term enrichment of their target genes revealed a strong E6-specific signature, with Fosl2 most prominently linked to keratinocyte development.

To determine which TFs identified by motif enrichment are also transcriptionally active in our cells, we intersected WGBS-derived DMR motifs with RNAseq data. This analysis identified a defined subset of TFs that were both expressed and associated with DMRs, representing the most likely functional regulators of methylation-driven transcriptional changes (Supplementary Table 7). Functional TF-GO associations revealed a striking E6-specific signature: Fosl2 showed the strongest and most selective association with the top enriched biological process, “keratinocyte development”, identifying it as the predominant regulator of differentiation pathways in E6-expressing cells. In contrast, E7 cells displayed a more diffuse TF-GO pattern but highlighted NF1 as the TF most strongly associated with the significantly enriched GO term “long-term synaptic potentiation”. E6/E7-expressing keratinocytes display TF–GO term association patterns that partially overlap with both the E6-and E7-associated signatures (Fig. 6). Taken together, these results show that HPV8 oncoproteins reshape the DNA methylation landscapes in manners that suggest altered redistribution of TF-associated regulatory regions. The convergence of these epigenetically regulated pathways on keratinocyte biology suggests a central mechanism by which HPV8 may promote a permissive environment for epithelial transformation.

To directly link DNA methylation changes to functional gene regulation, we mapped DMRs to the target genes of expressed TFs in each HPV8 condition. Most DMR-TF associations occurred within hypomethylated gene-body DMRs, which typically corresponded with increased expression, indicating that HPV8 enhances TF binding and transcriptional activation. In E6 cells, Fosl2 regulated targets were linked to hypomethylated gene bodies showing coherent transcriptional upregulation, consistent with E6-mediated enhancement of Fosl2 activity and its role in controlling keratinocyte differentiation (Supplementary Fig. 4; Supplementary Table 8).

Together, these findings demonstrate that HPV8 oncoproteins remodel the methylome in TF-specific and pathway-specific manners, establishing an E6-Fosl2 axis of epigenetic regulation. This coordinated reorganization of TF binding site accessibility and downstream gene expression reveals how HPV8 reshapes the epigenetic architecture of keratinocytes creating a cellular state permissive for epithelial transformation.

### 3.6 BetaHPV positivity in cutaneous lesions correlates with stem cell-like methylation profiles

**Fig. 7:**
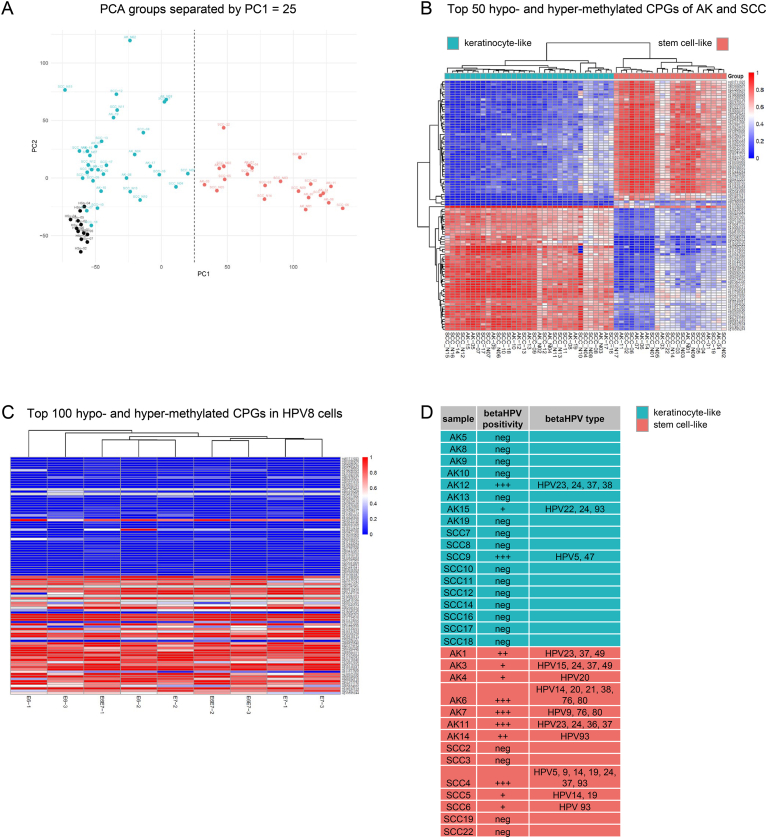
BetaHPV positivity correlates with stem cell-like methylation profiles. (A) Unsupervised comparison of DNA methylation profiles of N/TERT keratinocytes expressing HPV8-E6, -E7, or E6/E7 with published methylation datasets from benign warts [31]. Samples cluster according to keratinocyte-like methylation signatures under culture conditions used. (B) Projection of HPV8 oncogene–expressing N/TERT keratinocytes onto reference epidermal methylation states derived from the wart dataset, confirming that cultured cells retain keratinocyte-like rather than stem-cell–like (Epi-SC) methylation profiles. (C) Comparison of methylation profiles of N/TERT keratinocytes expressing HPV8-E6, E7, or E6/E7 with published datasets from benign warts [31] revealed keratinocyte-like signatures under N/TERT culture conditions. (D) Actinic keratoses (AK) and cutaneous squamous cell carcinomas (cSCC) with known methylation profiles [30] were tested for betaHPV DNA. Lesions with stem Epi-SC-like methylation profiles were significantly more often betaHPV-positive than those with keratinocyte-like profiles (69% vs. 22%).

Having established that HPV8 oncoproteins reshape the methylome through TF-specific mechanisms that reprogram keratinocyte differentiation pathways, we next asked whether these epigenetic alterations also influence the broader cellular identity of HPV-expressing keratinocytes. Specifically, we investigated whether the resulting methylation profiles reflect distinct keratinocyte sub-lineages, such as Epi-SC-like or keratinocyte-like states. To address this, we compared the methylation profiles of our N/TERT keratinocytes expressing HPV8-E6, -E7, or -E6/E7 with published datasets from benign warts caused by low-risk cutaneous HPV types [31]. Similar to common warts, which exhibit keratinocyte-like methylation profiles, HPV8 oncogene-expression in N/TERT cells also resulted in keratinocyte-like signatures under the culture conditions used in the present study (Fig. 7A, B, C).

We next investigated whether the two distinct methylation subtypes of AK and cSCC, which likely originate from different keratinocyte differentiation stages, differ in their association with betaHPV DNA positivity. To this end, previously published AK and cSCC samples with defined methylation profiles [30] were subjected to betaHPV-specific PCR followed by genotyping. AK and cSCC lesions with an Epi-SC-like methylation profile were more frequently betaHPV-positive than those with keratinocyte-like profiles. In the Epi-SC-like group, 9 of 13 lesions (69%) were betaHPV-positive, including several with multiple virus types and high viral loads. By contrast, in the keratinocyte-like group, only 4 of 18 lesions (22%) were betaHPV-positive, mostly harboring single virus types or low viral loads (Fig. 7D).

## 4 Discussion

Our study provides an integrated analysis of how HPV8 oncoproteins reshape keratinocyte identity through coordinated transcriptional and epigenetic mechanisms. By combining RNAseq, small RNA profiling, and WGBS, we demonstrate that HPV8 - particularly the E6 oncoprotein - acts as a dominant regulator of host gene expression and epigenetic regulator in keratinocytes. Compared with E7, E6 induced extensive gene expression changes, characterized by repression of epidermal differentiation pathways. The transcriptional profile of E6/E7-expressing cells largely mirrored that of E6 alone, underscoring that E6 dictates the overall regulatory architecture when both oncoproteins are co-expressed. Importantly, the reduced number of DEGs observed in E6/E7 cells was not caused by lower E6 transcript levels as E6 expression levels were comparable between E6 and E6/E7 cells, suggesting functional modulation of E6-driven transcriptional programs by E7 co-expression.

These findings align with transgenic mouse models in which HPV8-E6 drives epidermal hyperplasia, stem cell expansion, and tumor susceptibility [7,43]. The repression of lineage-stabilizing TFs such as OVOL1, GRHL3, and KLF4, together with downregulation of desmosomal and barrier-associated genes (DSG1, SPRR1B), indicates that E6 disrupts core differentiation circuits that maintain epidermal homeostasis. Such destabilization of lineage identity may create a cellular environment favorable for the viral life cycle and may also lower the threshold for malignant transformation in the presence of additional oncogenic stressors such as UV exposure.

Increasing evidence has shown that miRNAs regulate the expression of mRNAs involved in key signaling pathways that control cancer stem cell maintenance and differentiation [44]. In addition to transcriptional reprogramming, our data identify miRNA remodeling as an additional regulatory layer contributing to HPV8-driven gene expression changes. Integrative analyses revealed reciprocal enrichment patterns consistent with functional miRNA–target relationships, supporting a direct contribution of deregulated miRNAs to the observed mRNA expression changes. Among the downregulated miRNAs, the let-7a-5p hub is notable because members of the let-7 family are key regulators of epithelial differentiation and stemness [45]. Reduced let-7 activity has been linked to derepression of oncogenic targets and activation of RAS–MAPK signaling, promoting proliferative and progenitor-like transcriptional states [46]. Enhanced RAS–MAPK signaling can in turn stimulate AP1 transcription factors, which is notable in the context of our data where AP1 motifs and Fosl2-associated regulatory networks emerged as prominent features of the methylation-linked transcriptional landscape [47]. Together, these observations are consistent with the repression of differentiation programs observed in HPV8-E6-expressing keratinocytes and support a model in which downregulation of let-7a-5p is consistent with E6-driven destabilization of keratinocyte lineage identity. Network analysis also identified multiple upregulated miRNAs whose predicted targets are enriched among the downregulated transcripts, suggesting that miRNA-mediated repression contributes directly to the transcriptional changes observed in HPV8-E6–expressing keratinocytes. These findings are further supported by our previous work demonstrating that HPV8 modulates host miRNA expression during tumor development in HPV8 transgenic mice, where the expression of several miRNAs was found to be modified in skin tumors arising in HPV8-E6–expressing animals [48]. These observations reinforce the concept that HPV8-driven epithelial transformation involves alterations of miRNA regulatory networks in vivo.

WGBS revealed widespread redistribution of DNA methylation, affecting promoters, enhancers, and gene bodies. The predominance of DMRs in E6-expressing cells, and their strong overlap with E6/E7 profiles, further reinforces the dominant role of E6 in epigenetic remodeling. These methylation changes were not confined to isolated loci but involved large-scale alterations in partially methylated domains and regulatory elements, indicating global restructuring of the keratinocyte methylome. Together, these data support a multilayered model in which HPV8-E6 reshapes TF activity, adjusts miRNA-mediated tuning and modifies DNA methylation landscapes to establish a stably reprogrammed cellular state, favorable for virus replication.

By integrating transcriptional data with methylation landscapes, we show that HPV8 oncoproteins exert multilayered control over transcription factor networks by reshaping TF motif landscapes associated with regulatory accessibility and the expression of their downstream targets. Notably, motif enrichment analysis revealed a strong overrepresentation of AP-1–related binding motifs, consistent with the AP-1–associated regulatory signatures already suggested by our miRNA analyses. Among the regulators emerging from this analysis, Fosl2 stands out as a functionally relevant TF whose predicted target genes display hypomethylated gene-body DMRs or hypermethylated enhancer DMRs in E6-expressing keratinocytes. This DMR signature positions Fosl2 as a key mediator of the differentiation-associated programs highlighted in our TF-GO term analysis. Given the established role of Fosl2 in epidermal differentiation and barrier formation, modulation of Fosl2-associated circuits may represent a key mechanism by which E6 perturbs keratinocyte lineage commitment as part of a broader epigenetic rewiring of keratinocyte identity [49]. In addition, AP1 family activation has been implicated in cutaneous carcinogenesis, raising the possibility that E6-mediated reconfiguration of AP1/Fosl2 networks contributes both to early viral persistence and long-term oncogenic potential [50]. While our data identify a strong association between methylation changes, motif redistribution, and Fosl2-linked gene expression, functional validation will be required to determine whether direct modulation of Fosl2 activity can rescue differentiation defects in HPV8-E6-expressing keratinocytes. In contrast to E6, E7 induced more limited and selective transcriptional and methylation changes. Motif analysis highlighted distinct regulatory signatures, including associations with NF1-linked pathways. These findings suggest that E7 does not globally reprogram keratinocyte identity but rather modulates specific signaling modules that may cooperate with E6-driven lineage destabilization. Thus, HPV8 oncogene cooperation appears functionally asymmetric, with E6 establishing the primary epigenetic framework and E7 refining selected regulatory circuits.

A limitation of our study is that a mycoplasma contamination was retrospectively detected in the HPV8-E6 RNAseq samples after completion of sequencing analyses. Although mycoplasma contamination can affect cellular transcriptional programs, several observations indicate that the principal conclusions of this study are robust. First, independent validation experiments performed in mycoplasma-free HPV8-E6 keratinocytes reproduced the repression of differentiation-associated genes identified by RNAseq. Second, the independently maintained and continuously mycoplasma-negative HPV8-E6/E7 cells consistently recapitulated the major E6-associated transcriptomic, miRNA, and methylation signatures across all omics layers. Finally, the observed enrichment of structured differentiation-associated and AP1/Fosl2-linked regulatory programs is not typical of generalized mycoplasma-induced stress responses. Nevertheless, we cannot fully exclude that the contamination may have contributed to the magnitude of selected transcriptional alterations in the original E6 dataset.

Extending our molecular findings to human disease, we demonstrate that betaHPV DNA positivity is significantly enriched in actinic keratoses and cSCCs displaying Epi-SC-like methylation profiles. In contrast, lesions with keratinocyte-like methylation signatures were less frequently betaHPV-positive. This association supports a model in which betaHPVs preferentially act in, or impact, progenitor or stem cell compartments during early tumorigenesis. Although HPV8-expressing N/TERT keratinocytes under standard culture conditions retained keratinocyte-like methylation patterns, repression of differentiation factors and activation of stemness-associated pathways by E6, e.g. via STAT3 and ΔNp63 [12] are consistent with partial lineage destabilization. The absence of a full stem cell–like methylation phenotype in vitro likely reflects the lack of microenvironmental cues present in vivo. Taken together, these observations connect betaHPV activity with a clinically defined epigenetic tumor subclass and suggest that betaHPV-associated carcinogenesis preferentially engages specific epidermal cell hierarchies.

Together, our integrated analyses support a model in which HPV8-E6 acts as the principal driver of coordinated transcriptional and epigenetic remodeling in keratinocytes. E6 induces extensive alterations in protein-coding gene and miRNA expression, reshapes DNA methylation landscapes at regulatory elements, and is associated with redistribution of TF associated regulatory networks. Collectively, these multilayered changes are consistent with destabilization of epidermal differentiation programs and may contribute to the establishment of a more plastic cellular state that could favor viral persistence and increase susceptibility to malignant progression. In contrast, E7 contributes more selective and complementary regulatory alterations but does not override the dominant E6-associated transcriptional and epigenetic framework. Although our multi-omics analyses identify coherent regulatory programs associated with altered keratinocyte identity, additional functional studies, including functional validation of TF occupancy analyses as well as validating key miRNAs, will be required to directly determine the consequences of these changes for differentiation and transformation processes. In summary, our study reveals that HPV8 oncoproteins - predominantly E6 - induce coordinated transcriptional and epigenetic remodeling in keratinocytes. The enrichment of betaHPV in subclasses of AK and cSCC with Epi-SC-like methylation profiles further underscores the clinical relevance of methylation-linked reprogramming. These findings provide a comprehensive framework for understanding how betaHPVs contribute to early events in cutaneous carcinogenesis.

## Declaration of generative AI and AI-assisted technologies in the manuscript preparation process

During the preparation of this work the author(s) used ChatGPT (v.5) for proof-reading and grammar correction. After using this tool, the authors reviewed and edited the content as needed and take full responsibility for the content of the published article.

## Funding

This study was funded by the Deutsche Forschungsgemeinschaft (DFG, German Research Foundation, grant number 411052531 and 502233329 to BA) and by a grant from the Cancer Foundation Finland (to EA).

## CRediT authorship contribution statement

**Martin Hufbauer:** Data curation, Formal analysis, Investigation, Methodology, Validation, Visualization, Writing – original draft. **Adnan Syed:** Data curation, Investigation, Visualization. **Felix Bormann:** Conceptualization, Data curation, Formal analysis, Investigation, Methodology, Software, Validation, Visualization, Writing – original draft. **Daniel Hasche:** Formal analysis, Investigation, Writing – original draft. **Manuel Rodríguez-Paredes:** Formal analysis, Methodology, Visualization, Writing – original draft. **Pia Laine:** Data curation, Formal analysis, Investigation, Methodology, Validation, Writing – original draft. **Anni Honkimaa:** Formal analysis, Investigation, Visualization, Writing – original draft. **Petri Auvinen:** Conceptualization, Data curation, Formal analysis, Investigation, Methodology, Resources, Supervision, Validation, Visualization, Writing – original draft. **Eeva Auvinen:** Conceptualization, Data curation, Formal analysis, Funding acquisition, Investigation, Methodology, Project administration, Resources, Supervision, Validation, Visualization, Writing – original draft. **Baki Akgül:** Conceptualization, Data curation, Formal analysis, Funding acquisition, Investigation, Methodology, Project administration, Resources, Supervision, Validation, Visualization, Writing – original draft.

## Declaration of competing interest

The authors declare the following financial interests/personal relationships which may be considered as potential competing interests:Baki Akgül reports financial support was provided by German Research Foundation. Eeva Auvinen reports financial support was provided by Cancer Foundation Finland. If there are other authors, they declare that they have no known competing financial interests or personal relationships that could have appeared to influence the work reported in this paper.

## Data Availability

All data are included in the manuscript and Supplementary Information. Sequencing data are available in the European Nucleotide Archive (ENA) under accession PRJEB108515.

## Appendix A Supplementary data

Supplementary Figure 1 Alignment of HPV8 oncogene specific transcripts. (A) Linear map of the HPV8 genome taken from the PaVE database (pave.niaid.nih.gov, [51]). (B) RNA-seq reads from HPV8-E6, -E7, -E6/E7 and control cells mapped to the HPV8 genome (accession number M12737).

Supplementary Figure 2 Mycoplasma contamination does not affect transcriptional conclusions. During data analysis, a mycoplasma infection was detected in HPV8-E6-expressing cells. RT-qPCR validation confirmed that global transcription patterns remained unaffected, demonstrating that the conclusions are robust despite the contamination. Bars represent mean expression ± SD of three biological replicates measured in duplicate (E6 M+ = mycoplasma-contaminated; E6 M − = mycoplasma-free E6 cells).

Supplementary Figure 3 WGBS quality control metrics. (A) Bisulfite conversion efficiency for all samples expressing HPV8-E6, -E7, -E6/E7 and vector control cells. Conversion rates were consistently high across libraries, indicating efficient bisulfite treatment. (B) Empirical cumulative distribution function (ECDF) plot showing CpG coverage across all samples. Similar coverage distributions indicate comparable sequencing depth and CpG representation among conditions. (C) Distribution of global CpG methylation levels across samples, demonstrating comparable overall methylation profiles between experimental groups and confirming suitability for downstream differential methylation analysis.

Supplementary Figure 4 Visualization of DMRs associated with TF-target genes in E6 and E7 cells. Smoothed methylation profiles for control (black curves) and HPV8-E6 or HPV8-E7 cells (blue curves) are shown across genomic regions containing DMRs linked to Fosl2 (E6) or NF1 (E7). The pink-shaded area marks the DMR, and black ticks indicate CpG positions. The lower panels display nearby annotated genes, with the Fosl2-or NF1-target gene highlighted in bold.

Supplementary Table 1 List of primers used for RT-qPCR.

Supplementary Table 2 Differentially expressed genes in HPV8 oncogene-expressing keratinocytes. RNAseq analysis of N/TERT keratinocytes expressing HPV8-E6, E7, or E6/E7 compared with empty vector transduced cells. The table lists log2 fold changes and adjusted p-values (padj). Separate sheets provide gene lists for HPV8-E6, -E7, and - E6/E7. The significantly deregulated genes (|log2FC| > 1; padj <0.05) and an overlap of these genes are listed in separate sheets.

Supplementary Table 3 Gene Ontology (GO) enrichment analysis of differentially expressed genes. GO biological process terms enriched among upregulated and downregulated genes in HPV8-E6, -E7, or -E6/E7 expressing cells compared to controls. Separate sheets list significantly enriched pathways (FDR <0.05) for each condition, including the number of genes per category, enrichment scores, and adjusted p-values.

Supplementary Table 4 miRNA identifiers and changes identified by small RNA sequencing.

Supplementary Table 5 DMRs identified in HPV8 oncogene-expressing keratinocytes. WGBS of N/TERT keratinocytes expressing HPV8-E6, -E7 or -E6/E7 identified genomic regions with significant methylation changes relative to controls (absolute methylation ≥0.15). Listed are chromosomal coordinates, mean methylation differences, statistical significance and direction of methylation (hyper- or hypomethylated DMRs). Separate sheets report comparison of DMRs between different HPV8 oncogene conditions and controls.

Supplementary Table 6 TF binding motif enrichment in DMRs. HOMER motif enrichment analysis of hyper- and hypomethylated DMRs identified by WGBS in HPV8-E6, -E7 or -E6/E7 expressing cells compared to empty vector controls. For each condition and methylation category, enriched motifs are listed together with their associated TF families and Homer calculated q-values. The TF binding motif enrichment for promoter, enhancer or gene body in each oncogene condition are listed in separate sheets.

Supplementary Table 7 Evaluation of the top 20 TFs with enriched binding sites. These TFs represent functional regulators of methylation-driven transcriptional changes.

Supplementary Table 8 Lists of genes linked to DMRs associated with TF motifs identified in HOMER analyses. Separate sheets provide results for HPV8-E6, HPV8-E7, and HPV8-E6/E7 conditions, stratified by hyper- and hypomethylated regions. Each entry includes TF, genomic location of DMR (promoter, enhancer, or gene body), methylation status, and corresponding target genes.
