# Supplementary figures and images for: HPV8-E6 drives coordinated transcriptional and epigenetic reprogramming of keratinocytes
Source: Tumour Virus Res. 2026 Jul 13;22:200348. doi: 10.1016/j.tvr.2026.200348 (PMC13383346; doi:10.1016/j.tvr.2026.200348)

## Slide 1
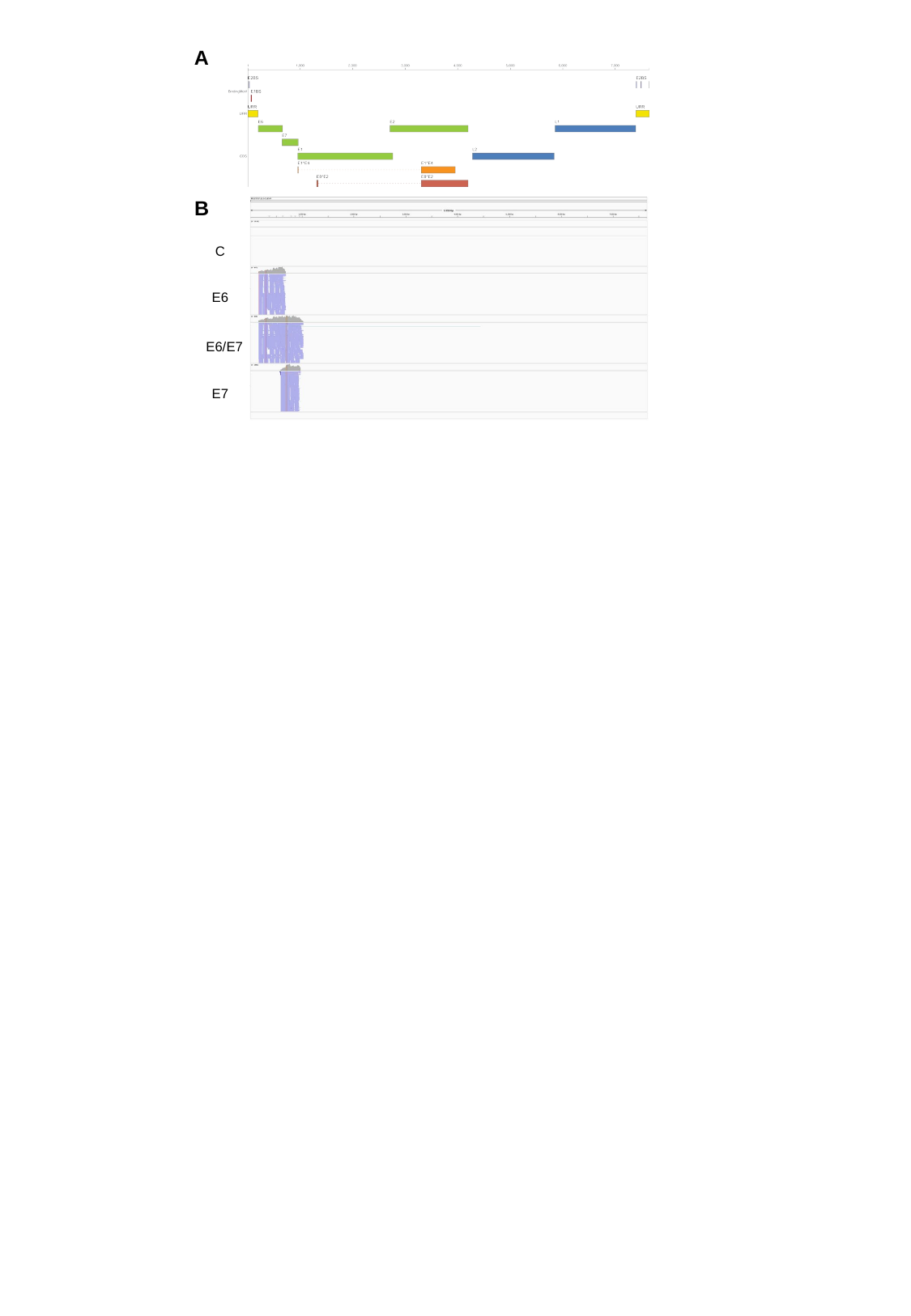

A
B
C
E6
E6/E7
E7

Supplement: Supplementary Figure 1 — Alignment of HPV8 oncogene specific transcripts. (A) Linear map of the HPV8 genome taken from the PaVE database (pave.niaid.nih.gov, [51]). (B) RNA-seq reads from HPV8-E6, -E7, -E6/E7 and control cells mapped to the HPV8 genome (accession number M12737). [file mmc1.pptx]

## Slide 1
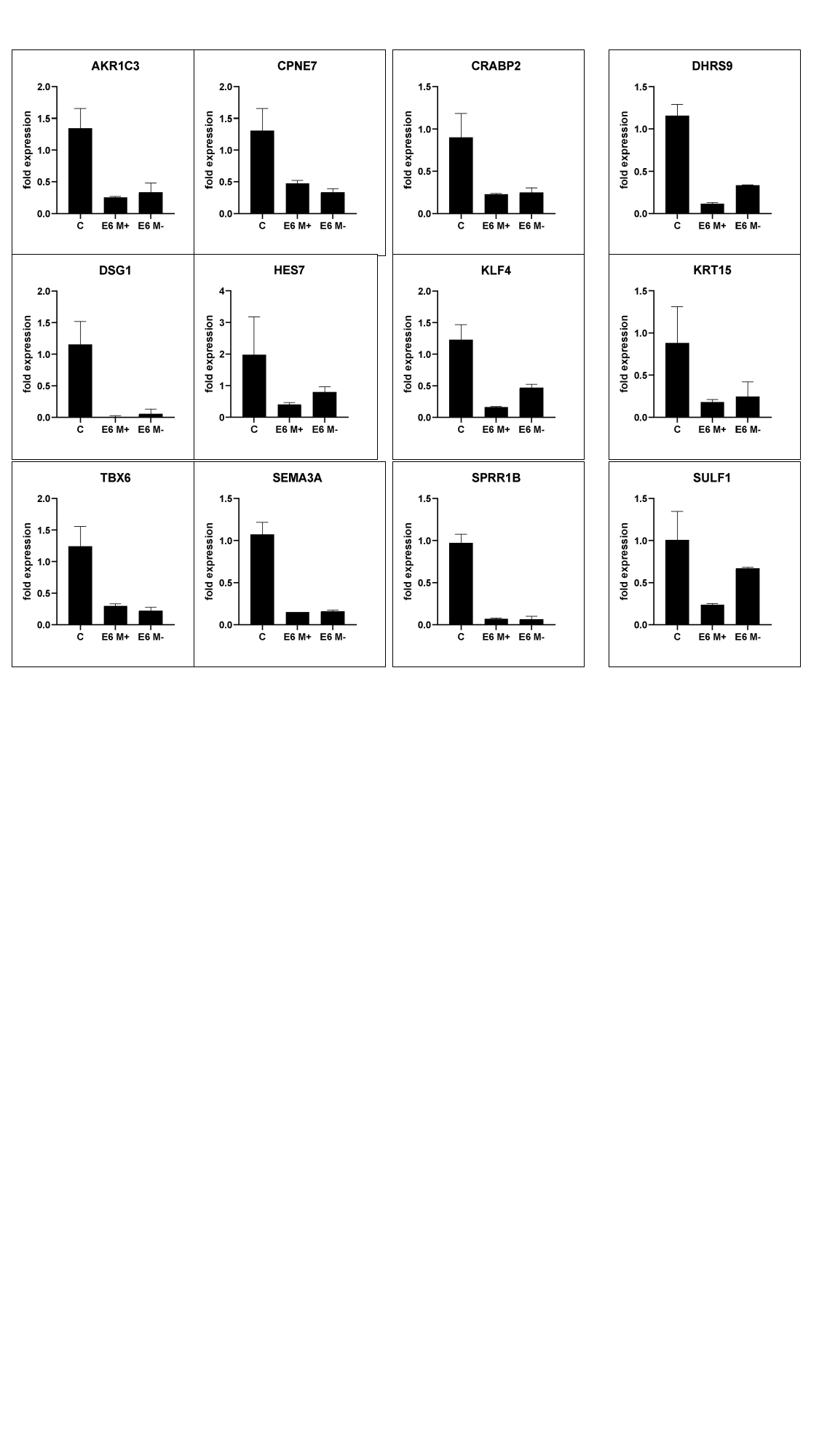

Supplement: Supplementary Figure 2 — Mycoplasma contamination does not affect transcriptional conclusions. During data analysis, a mycoplasma infection was detected in HPV8-E6-expressing cells. RT-qPCR validation confirmed that global transcription patterns remained unaffected, demonstrating that the conclusions are robust despite the contamination. Bars represent mean expression ± SD of three biological replicates measured in duplicate (E6 M+ = mycoplasma-contaminated; E6 M − = mycoplasma-free E6 cells). [file mmc2.pptx]

## Slide 1
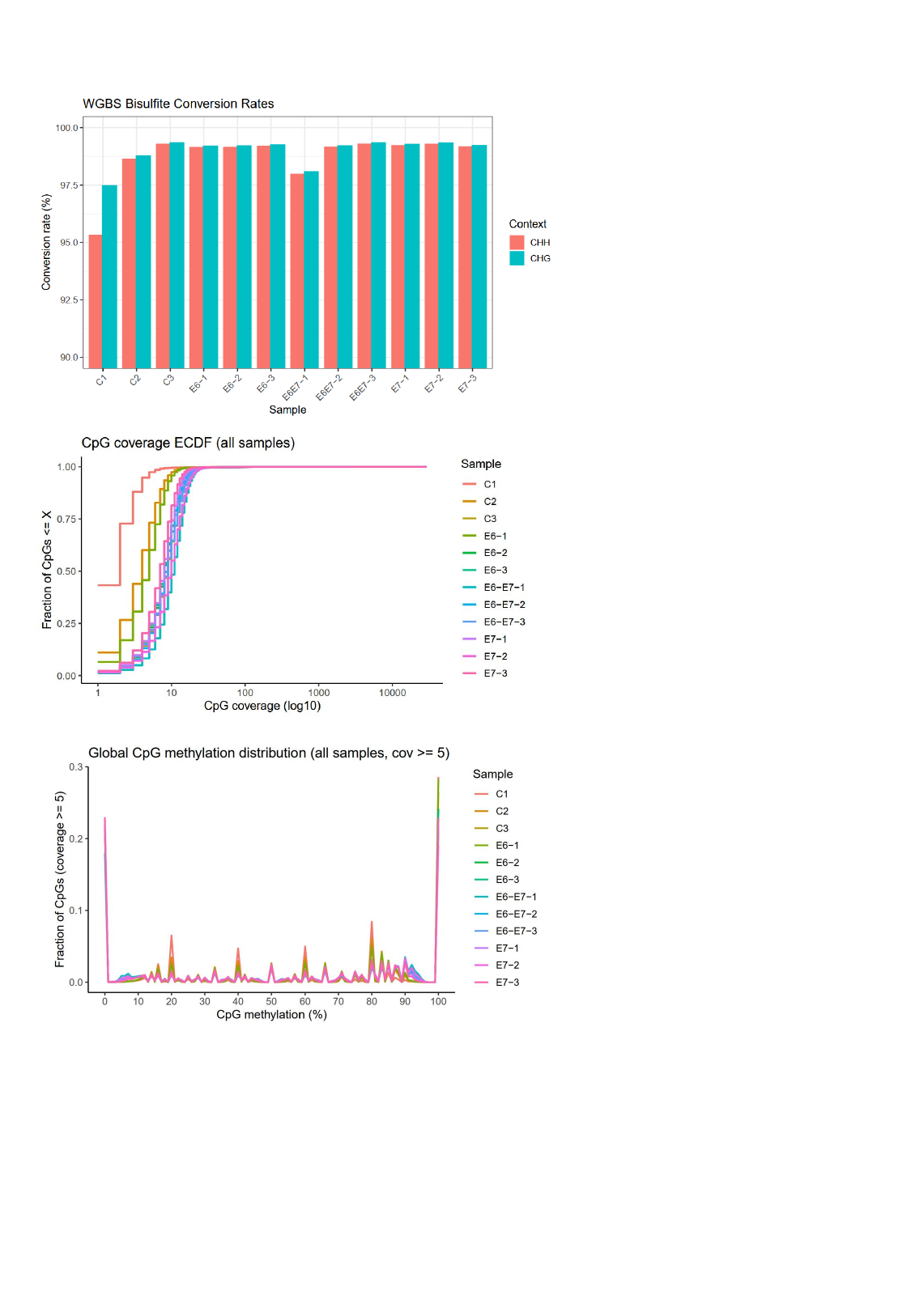

Supplement: Supplementary Figure 3 — WGBS quality control metrics. (A) Bisulfite conversion efficiency for all samples expressing HPV8-E6, -E7, -E6/E7 and vector control cells. Conversion rates were consistently high across libraries, indicating efficient bisulfite treatment. (B) Empirical cumulative distribution function (ECDF) plot showing CpG coverage across all samples. Similar coverage distributions indicate comparable sequencing depth and CpG representation among conditions. (C) Distribution of global CpG methylation levels across samples, demonstrating comparable overall methylation profiles between experimental groups and confirming suitability for downstream differential methylation analysis. [file mmc3.pptx]

## Slide 1
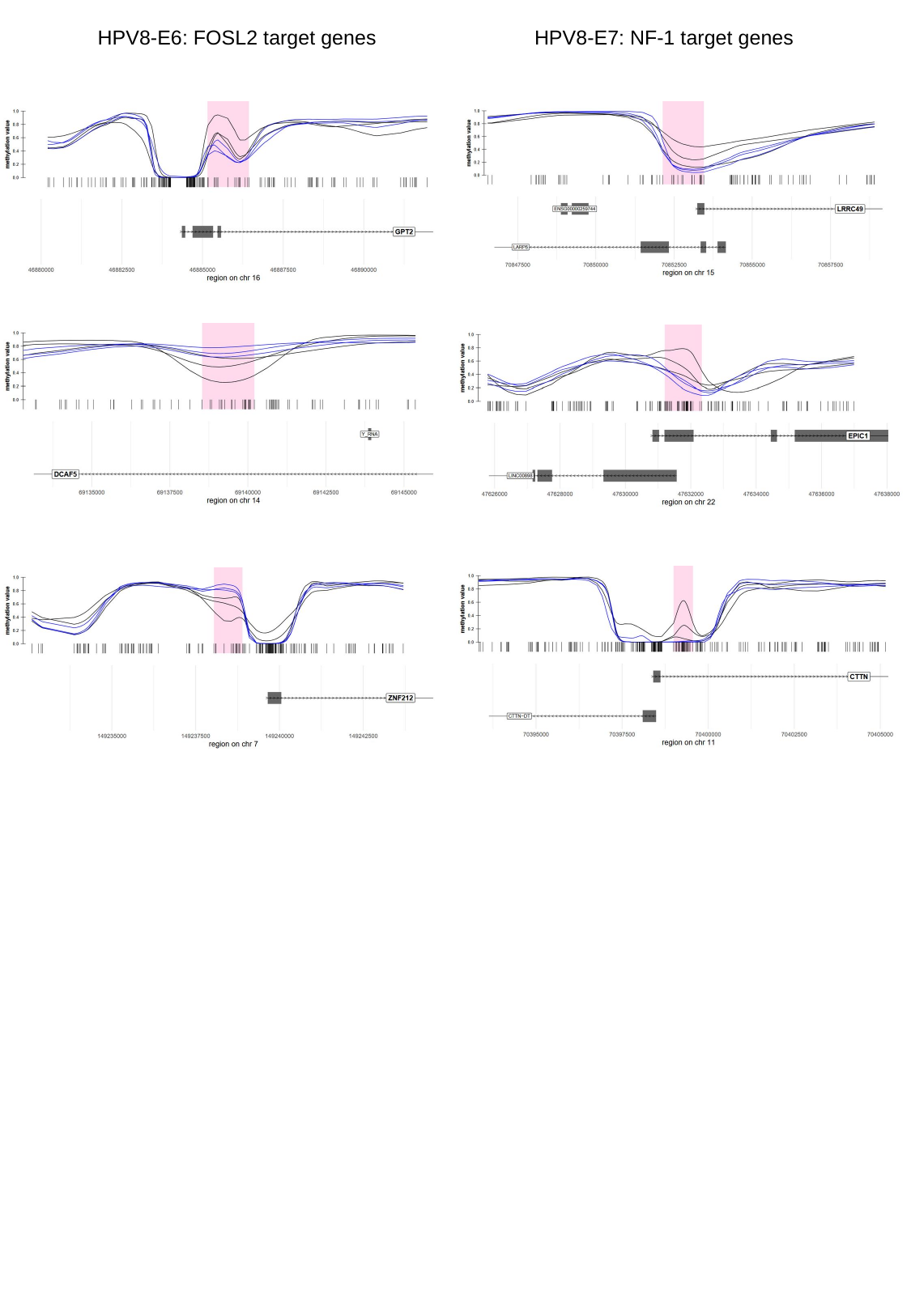

HPV8-E6: FOSL2 target genes
HPV8-E7: NF-1 target genes

Supplement: Supplementary Figure 4 — Visualization of DMRs associated with TF-target genes in E6 and E7 cells. Smoothed methylation profiles for control (black curves) and HPV8-E6 or HPV8-E7 cells (blue curves) are shown across genomic regions containing DMRs linked to Fosl2 (E6) or NF1 (E7). The pink-shaded area marks the DMR, and black ticks indicate CpG positions. The lower panels display nearby annotated genes, with the Fosl2-or NF1-target gene highlighted in bold. [file mmc4.pptx]
